# The upper respiratory tract microbiome and its potential role in bovine respiratory disease and otitis media

**DOI:** 10.1038/srep29050

**Published:** 2016-07-01

**Authors:** Svetlana F. Lima, Andre Gustavo V. Teixeira, Catherine H. Higgins, Fabio S. Lima, Rodrigo C. Bicalho

**Affiliations:** 1 Department of Population Medicine and Diagnostic Sciences, College of Veterinary Medicine, Cornell University, Ithaca, New York, USA; 2 Department of Veterinary Clinical Medicine, College of Veterinary Medicine, University of Illinois, Urbana, Illinois, USA

## Abstract

The upper respiratory tract (URT) hosts a complex microbial community of commensal microorganisms and potential pathogens. Analyzing the composition and nature of the healthy URT microbiota and how it changes over time will contribute to a better understanding of the pathogenesis of pneumonia and otitis. A longitudinal study was conducted including 174 Holstein calves that were divided in four groups: healthy calves, calves diagnosed with pneumonia, otitis or both diseases. Deep pharyngeal swabs were collected on days 3, 14, 28, and 35 of life, and next-generation sequencing of the 16S rRNA gene as well as quantitative PCR was performed. The URT of Holstein dairy calves aged 3 to 35 days revealed to host a highly diverse bacterial community. The relative abundances of the bacterial genera Mannheimia, Moraxella, and Mycoplasma were significantly higher in diseased versus healthy animals, and the total bacterial load of newborn calves at day 3 was higher for animals that developed pneumonia than for healthy animals. Our results corroborate the existing knowledge that species of Mannheimia and Mycoplasma are important pathogens in pneumonia and otitis. Furthermore, they suggest that species of Moraxella can potentially cause the same disorders (pneumonia and otitis), and that high neonatal bacterial load is a key contributor to the development of pneumonia.

Bovine respiratory disease (BRD) is a complex, multifactorial disorder caused by a combination of microbial pathogens^1^,^2^,^3^, impaired host immunity^4^,^5^,^6^, environmental factors^5^,^6^,^7^, and inadequate housing conditions^5^,^6^. Despite advances in veterinary medicine and technology to control BRD, it remains a huge economic burden for both the dairy and beef industries due to calf mortality, treatment expenses, and additional labor incurred. Furthermore, BRD has substantial long-term consequences on performance by negatively impacting growth^8^, reproductive performance^9^, and longevity^9^,^10^. In the USA dairy industry, BRD is a major contributor to mortality and morbidity^11^. The United States Department of Agriculture National Animal Health Monitoring Service (NAHMS, 2007) reported that BRD affects 12.4% of calves during the pre-weaning period and is responsible for 22.5% of the mortality documented during the same period^11^. Additionally, 5.9% of post-weaning animals are eventually diagnosed with BRD, which is responsible for 46.5% of the mortality documented during the same period. The detrimental economic impact of BRD on the American beef industry is even larger than on the dairy industry. BRD is considered to be the most expensive disease affecting feedlot cattle and it has been estimated to cause losses of approximately one billion dollars per year in the USA^12^,^13^.

Several viral and bacterial etiological agents have been associated with bovine respiratory tract (RT) disease. Bovine viral diarrhea virus (BVDV), bovine respiratory syncytial virus (BRSV), and parainfluenza type 3 virus (PI-3) have all been described as important causative agents of the BRD complex^13^,^14^. It is thought that primary viral infection may render the RT epithelium more susceptible to bacterial colonization. Viral infections can impair the epithelial layer of the RT mucosa by disarranging host cellular functions and/or killing infected epithelial cells, thus exposing the RT basement membrane. Furthermore, viruses may damage ciliated host cells, resulting in reduced mucociliary velocity (reduction of bacterial clearance) and thus leading to a compromised host immune response to secondary bacterial infection^15^,^16^,^17^. The key bacterial pathogens associated with bovine pneumonia include *Pasteurella multocida*^18^,^19^, *Mannheimia haemolytica*^18^,^20^, *Histophilus somni*^21^, *Mycoplasma bovis*^20^,^21^, and other *Mycoplasma* spp.^3^.

Another relatively common disease affecting dairy calves is otitis media. This infection of the middle ear manifests as head tilt and sometimes facial paralysis due to involvement of cranial nerves VII and VIII and peripheral vestibular structures^22^,^23^,^24^. A recent study evaluated the impact of BRD, diarrhea, arthritis, and otitis on mortality and carcass traits in white veal calves, and an increased mortality risk was found for otitis (Hazard ratio equal to 7.0)^25^. It has been suggested that otitis media and pneumonia may evolve from URT infection, because anatomically the nasopharynx area communicates with the nose cavity, the sinuses, the middle ears, and the larynx, and URT-resident microbes can be a source for lower respiratory tract infections^26^,^27^. This is also true for middle ear infections, as the nasopharynx is connected to the middle ear via the Eustachian tube^27^, thereby supporting a strong link between the URT and both otitis and pneumonia. Moreover, both diseases affect calves of the same age and share common risk factors, and the most dominant bacteria reportedly involved in the etiology of bronchopneumonia are also associated with otitis media^22^. Pathogens commonly associated with otitis media include *Mannheimia haemolytica*^23^, *Histophilus somni*^28^, *Mycoplasma* spp.^29^, *Mycoplasma bovis*^30^,^31^, *Pasteurella multocida*^22^,^32^, *Staphylococcus* spp.^23^,^33^, and *Streptococcus* spp.^32^.

Currently, intensive efforts have focused on understanding the composition and nature of bodily microbial populations in a balanced microbiome state, and how shifts in such microbial community structures impact the health of both humans^1^,^34^ and animals^35^,^36^. According to Bosch *et al.*^17^, imbalances of the upper respiratory tract (URT) ecosystem may result in invasion by and overgrowth of bacterial pathogens, leading to respiratory disease. A recent study by Homan *et al.*^37^ assessed the URT microbiome of feedlot cattle on the day of arrival and again 60 days later and found significant differences between the URT microbiomes at the two time points. Unfortunately, comparisons between healthy and sick animals were not evaluated in that study^37^. To our knowledge, the URT microbiome of dairy calves has not been previously investigated using modern microbiome techniques. The 16S rRNA gene library-based molecular strategy is a powerful approach for identifying members of a microbial community and quantifying their relative abundance while avoiding the limitations imposed by culture-dependent methods and biochemical approaches^38^,^39^. Furthermore, it is a rapid and cost-effective method for assessing bacterial diversity and is a useful tool for pathogen discovery and identification^40^.

Therefore, the goal of this study was to characterize longitudinally the URT microbiome of Holstein dairy calves by using high-throughput sequencing of the 16S rRNA gene. We aimed to compare the URT microbial communities of healthy and unhealthy subjects at each time point of our data collection.

## Results

### Descriptive data

In total, 174 calves were enrolled in this study. Of these, 37 (21.3%) were diagnosed with pneumonia, 62 (35.6%) with otitis, 11 (6.3%) with pneumonia-otitis combined, and 64 (36.8%) were healthy (Table 1). The average age at first diagnosis was 22.5 days for pneumonia, 24.0 days for otitis, and 19.7 days for pneumonia-otitis combined (Table 1).

### Sequencing results

We collected 696 deep nasal swab samples from 174 Holstein calves. Samples were collected at days 3, 14, 28, and 35 of life. All samples collected were used individually to assess the microbiome by amplification and next-generation sequencing of the V4 region of the 16s rRNA gene. A total of 5 sequencing runs were performed using the Miseq sequencer (Illumina, Inc., San Diego, CA) and theV2 chemistry kits (300-cycles); approximately 140 barcoded samples were sequenced on each run.

Sequences were filtered for size, quality, and for the presence of chimeras and the total post-quality control number of sequences used in the study were 63,638,904. The average coverage was 91,567, the SD was 58,425, and the range was 1,423 to 657, 375 numbers of reads per sample.

### Number of reads, richness and diversity indexes, and 16S rRNA gene copy numbers

**Figure 1:**
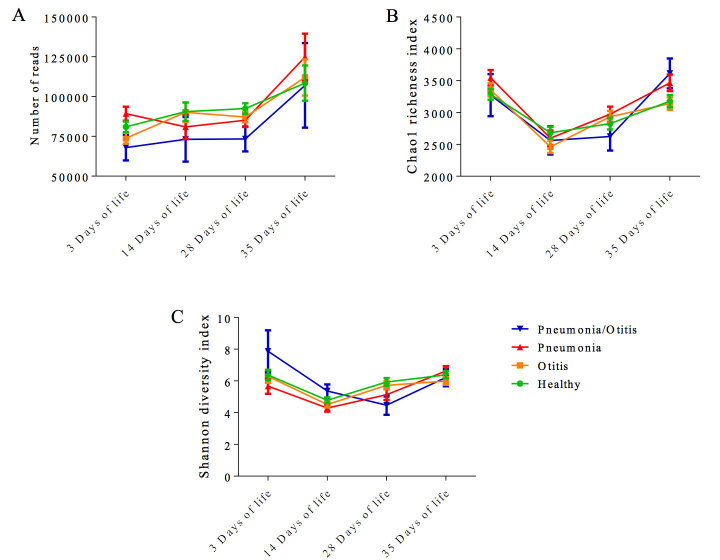
Bar graphs illustrating the number of reads (**A**), Chao 1 richness index (**B**) and Shannon diversity index (**C**) for different postnatal ages. Error bars represent standard errors. Dunnett’s multiple comparison procedure was used to compare each disease status (otitis, pneumonia, and pneumonia-otitis combined) against the status “healthy” within each sample collection date.

The mean number of reads for each health status (healthy, pneumonia, otitis, pneumonia-otitis combined) was not significantly different within each time point (Fig. 1A). Regarding OTU richness and diversity, the mean Chao1 richness index for each health status at each postnatal time point is illustrated in Fig. 1B. The mean Shannon diversity index for each health status at each postnatal time point is illustrated in Fig. 1C. Chao1 richness index is a nonparametric estimator of the minimum richness and is based on the number of rare OTUs (singletons and doublets), within a sample^41^. When a sample exhibits many singletons, it is likely that more undetected OTUs exist, and the Chao 1 richness index will estimate a higher richness than it would estimate for a sample deprived of rare OTUs. The Shannon diversity index accounts for both richness and abundance in a single value of evenness. Microbiomes that are numerically dominated by one or few organisms present low evenness, and when abundance is distributed equally among organisms the microbiome presents high evenness^42^. The richness and the evenness were analyzed to see whether any divergence is observed across health conditions. The Chao 1 and Shannon indexes did not differ significantly when comparing health statuses, regardless of the age time point (Fig. 1B,C).

A negative correlation was detected between the total bacterial load, as assessed by the number of 16S rRNA gene copies, and the Shannon diversity index (r = −0.40, *P*-value < 0.0001) (Supplemental 1). Similar negative correlations were found when data were stratified by the different disease statuses (Table 2). No correlation was found between the number of 16S rRNA gene copies and the Chao 1 richness index (r = −0.025, *P*-value = 0.20) (data not shown).

**Figure 2:**
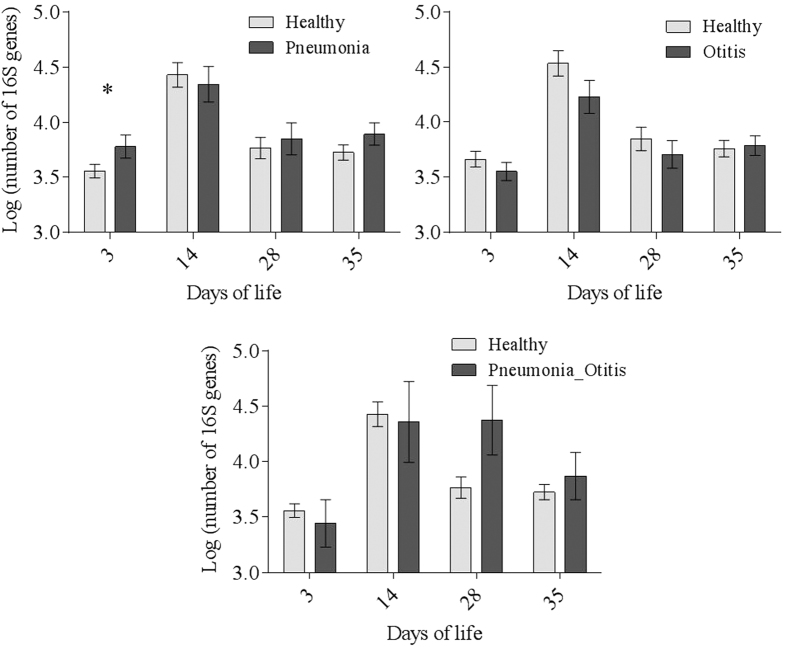
Mean log10 number of the 16S rRNA gene identified in upper respiratory tract samples of calves at various postnatal time points (3, 14, 28 and 35 days) and for different health statuses (healthy, pneumonia, and otitis). Dunnett’s multiple comparison procedure was used to compare each disease status (otitis, pneumonia, and pneumonia-otitis combined) against the status “healthy” within each sample collection date. An asterisk between health statuses represents a significant difference (*P* < 0.05) for the age sampled.

Quantitative real-time PCR was used to monitor the amplification of the 16s rRNA targeted gene during PCR. As a result of this method an absolute quantification that gives the exact number of the target DNA molecules within a sample, by comparison with DNA standards (serial dilution of our 16S rRNA gene clone) using a calibration curve is provided. At day 3 of life, healthy calves had significantly lower total bacterial loads, as defined by the log10 copy numbers of the 16S rRNA gene, than calves diagnosed with pneumonia. The average counts of the 16s rRNA gene copies at day 3 was 3.80 log_10_ (SE = 0.10) for animals that were later diagnosed with pneumonia and 3.55 log_10_ (SE = 0.06) for healthy animals (Fig. 2A).

### Microbial phylum analysis

**Figure 3:**
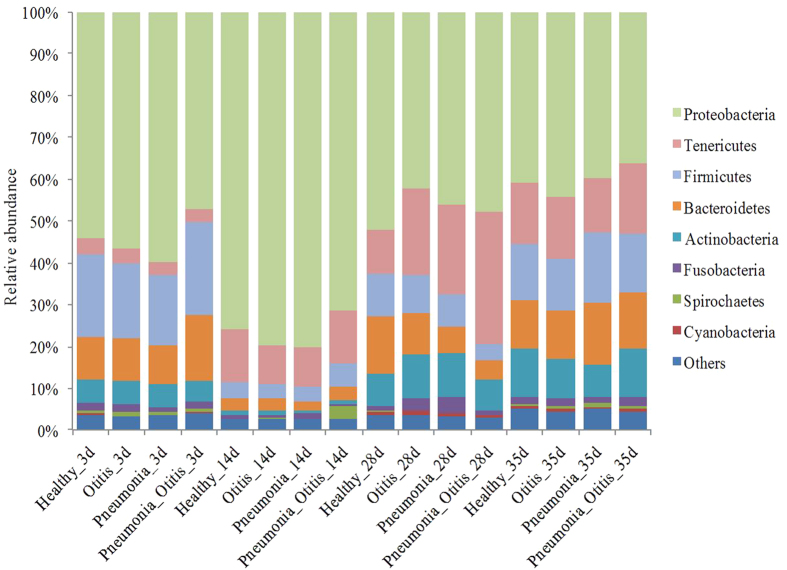
Mean relative abundance of the most prevalent bacterial phyla identified in upper respiratory tract samples of calves at various postnatal time points (3, 14, 28 and 35 days) and for different health statuses (healthy, pneumonia, otitis, and pneumonia-otitis combined).

The relative abundances of the eight most common phyla of the URT regardless of age and health conditions (Proteobacteria, Tenericutes, Firmicutes, Bacteroidetes, Actinobacteria, Fusobacteria, Spirochaetes, and Cyanobacteria) are depicted in Fig. 3. Proteobacteria was consistently the most abundant phylum across the four health categories (healthy, otitis, pneumonia, and pneumonia-otitis combined) (Fig. 3).

### Bacterial genus analysis

**Figure 4:**
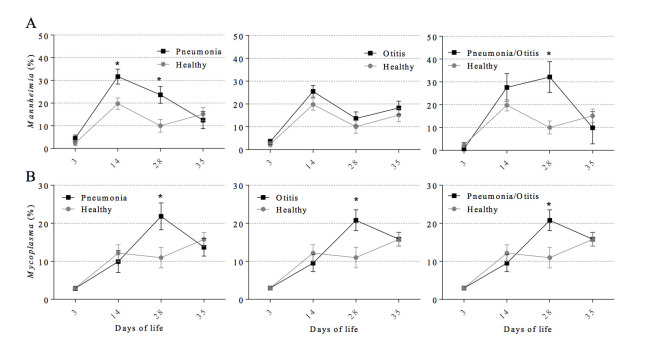
Mean relative abundance of the genus Mannheimia (A) and Mycoplasma (B) according to postnatal age at sample collection (3, 14, 28, 35 days) and health status (healthy, otitis, pneumonia, and pneumonia-otitis combined). Error bars are positioned around the means and represent the standard error of the mean. Dunnett’s multiple comparison procedure was used to compare each disease status (otitis, pneumonia, and pneumonia-otitis combined) against the status “healthy” within each sample collection time point. Asterisks on a series of data points indicate a significant difference (P < 0.05) between the respective health status categories within postnatal age.

The prevalence (% of animals detected with the respective OTU) of the 30 most abundant bacterial genera identified in the calf URT at days 3, 14, 24 and 35 of life is depicted in Table 3. The 30 most common bacterial genera and its respective mean relative abundance, identified throughout the different age time points and according to each health status evaluated are presented in Table 4. *Mannheimia, Mycoplasma, Moraxella, Psychrobacter,* and *Pseudomonas* were the top 5 genera regardless of health status (Table 4). Notably, *Mannheimia* and *Mycoplasma* were the bacterial genera with the greatest increase in relative abundance over time (Table 4 and Fig. 4A,B).

**Figure 5:**
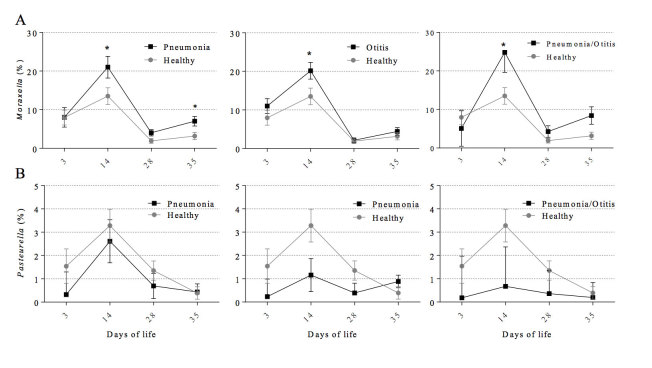
Mean relative abundance of the genus Moraxella (A) and Pasteurella (B) according to postnatal age at sample collection (3, 14, 28, 35 days) and health status (healthy, otitis, pneumonia, and pneumonia-otitis combined). Error bars are positioned around the means and represent the standard error of the mean. Dunnett’s multiple comparison procedure was used to compare each disease status (otitis, pneumonia, and pneumonia-otitis combined) against the status “healthy” within each sample collection time point. Asterisks on a series of data points indicate a significant difference (P < 0.05) between the respective health status categories within postnatal age.

The relative abundance of each of *Mannheimia* and *Moraxella* at day 14 in calves diagnosed with pneumonia was significantly higher when compared to healthy calves (Figs 4A and 5A). Similar results were observed for *Mannheimia* and *Mycoplasma* at day 28 (Fig. 4A,B). The relative abundance of *Pasteurella* at day 14 was lower in calves diagnosed with otitis when compared to healthy calves (Fig. 5B). The genera *Pseudomonas, Escherichia,* and *Corynebacterium* were also subjected to more detailed analyses and these are presented in supplemental material 2, 3, and 4.

## Discussion

To our knowledge, this is the first report to have longitudinally evaluated the URT microbiota of Holstein dairy calves from birth (day 3 of life) until 35 days of life. The URT is a critical point of entry for pathogens, and thus a potential route for infection of the lower respiratory tract^42^ and the middle ear^27^. Therefore, characterization of the URT is a crucial step in unraveling the pattern of development of both pneumonia and infection of the middle ear in calves. Here, we reported that the genera *Mannheimia, Moraxella*, and *Mycoplasma* were found in significantly higher abundances in dairy calves that developed pneumonia, otitis, or pneumonia-otitis combined during the pre-weaned period. We also observed that calves affected with pneumonia, otitis and pneumonia-otitis combined at day 3 of life presented the same bacterial community structure when compared to healthy animals, however animals diagnosed with pneumonia only at this same age had a significantly higher bacterial load, as defined by the log of the copy numbers of the 16S rRNA gene quantified by quantitative PCR technique, soon after they were born, than healthy calves. Together, these results suggest that the microbial composition of the URT of newborn calves (3 days of life) was not predictive of pneumonia, but the total URT bacterial load was. Therefore, pneumonia and otitis pathogens are already present in the URT of newborn calves, but those that will eventually develop respiratory disease simply have a higher load of total bacteria at 3 days of life.

The incidence of pneumonia and otitis reported in the present study was higher than the rates reported by the NAHMS 2002^43^ and 2007^11^; the reported incidence of BRD was 12.4% versus the 30% incidence herein reported. Our study was conducted on a commercial dairy farm that used a group house system (20 calves per group) and calves were fed ad-libitum acidified non-saleable milk. These types of systems have been reported to be associated with higher incidence of respiratory diseases such as pneumonia^44^. Svensson *et al.*^45^ evaluated the two types of pre-weaning housing systems, in which calves were raised in individual pens and milk was fed manually, or calves raised in grouping pens with automatic milk-feeding system^45^. Still in Svensson study, a higher odds ratios for respiratory disease and also increased respiratory sounds in calves housed in group pens with an automatic milk-feeding system (OR: 2.2, 2.8) than calves housed in individually pens was observed. Additionally, the increased chance of transmission of pathogenic agents between calves in housing group systems is also observed, since calves in this type of system tend to be more densely housed, resulting in closer animal-to-animal contact, and consequently propagation of the infections^46^,^47^,^48^. Therefore, this justifies the higher incidence rates reported in this present study.

In this present study, all calves that were diagnosed with pneumonia received antibiotic therapy. Systemic antibiotic therapy in calves has been reported to alter the fecal microbiome^49^ and most likely should also impact the microbiome of the URT. Therefore, the URT microbiome of post disease diagnostic here described has most likely been altered by the use of systemic antibiotics. Our study was conducted on a commercial dairy farm and farm protocols could not be modified solely for the purpose of the study. Additionally, pneumonia and otitis are diseases commonly caused by bacterial infection and neglecting to treat sick calves with the proper antibiotic therapy could be considered inhumane and may not have been approved by the Cornell Institutional Animal Care and Use Committee. Nevertheless, this is a limitation of the current study and future work should explore the URT microbiome for calves affected or not by respiratory diseases and treated or not by systemic antibiotic therapy.

An important result of the present study was the association between the genus *Moraxella* and, disease statuses. Calves that were diagnosed with pneumonia, otitis, or pneumonia-otitis combined had a significantly higher abundance of *Moraxella* at day 14 when compared to healthy calves. Although members of the genus *Moraxella* are often isolated from cases of keratoconjunctivitis, an important ocular illness in bovines^50^,^51^,^52^, there are few reports in the scientific literature describing an association of the genus *Moraxella* with pneumonia and/or otitis media. Catry *et al.*^53^ isolated *Moraxella ovis* from the upper and lower respiratory tract of calves affected with acute and chronic respiratory disease^53^, and Corbeil *et al.*^54^ reported that *Moraxella* spp. enhance the growth of RT bacterial pathogens such as *Mannheimia haemolytica*, *Pasteurella multocida,* and *Haemophilus somnus*^54^. The association between the genus *Moraxella* and the incidence of pneumonia and otitis in pre-weaned dairy cattle described in the present study is novel and of potential significance.

The abundance of *Mannheimia* and *Mycoplasma* increased substantially over time. At days 14, *Mannheimia* were significantly more abundant in disease statuses, and the same trend was observed for *Mannheimia* and *Mycoplasma* at day 28 of life. It is known that *Mannheimia haemolytica* is a commensal organism that inhabits the nasopharynx and can lead to disease when calves are exposed to stress factors such as weaning, comingling, and coinfection with others microorganisms^55^, such as *Mycoplasma bovis*^13^,^56^, which is suggested by ours results. Furthermore, *Pasteurella*, another important bacterium for the BRD complex in calves^18^,^19^,^57^, was not associated with disease nor was the genus highly abundant in any health status or age group in our analyses. Our data are supported by the results of Klima *et al.*, who detected a lower occurrence of *Pasteurella multocida* in a BRD outbreak in North American feedlots^58^.

In our study the most abundant genera detected in the URT of dairy calves were *Mannheimia, Mycoplasma, Moraxella, Psychrobacter,* and *Pseudomonas*. Recently, Holman *et al.*^37^ investigated the nasopharyngeal bacterial community of feedlot cattle at the day of feedlot entry and also at 60 days after entrance^37^. The Holman experiment focused on differences in the microbial community over time, and comparisons between healthy and diseased animals were not evaluated. Still, in the Holman study, *Staphylococcus*, *Mycoplasma*, *Mannheimia*, and *Moraxella* were the dominant genera identified in the URT samples collected at 60 days after feedlot arrival, comparable to what we observed in young calves with bacterial infections. The Holman results at 60 days may in part be a consequence of the high levels of stress (caused by shipping, change of environment, and stocking density)^59^ that beef calves experience following their arrival on the feedlot.

The genera *Mycoplasma, Mannheimia, Pasteurella, Staphylococcus,* and *Streptococcus* were detected in all animals at four time points examined, regardless of health conditions. These pathogens have been repeatedly described as the primary cause of otitis media^30^,^60^ and/or pneumonia^1^,^3^,^60^. A potential explanation for this finding could be that disease develops when host and/or pathogen factors result in bacterial proliferation and dissemination to other body sites^56^, and/or as a result of a detrimental host inflammatory response^26^,^61^. Further studies are required to examine both the microbial composition of the RT and the relative contribution of the immune system to the calf’s RT health.

The microbial diversity is understood as being a function of the number of different categories (richness) and the relative distribution of individual elements among these categories (evenness)^62^. Shannon diversity and Chao 1 richness indexes were not significantly different between healthy calves and calves diagnosed with BRD. The high microbial diversity detected in our experiment was expected since the URT is the first compartment of the respiratory system that is in close contact with the environment. In agreement with our findings, Charlson *et al.*^63^ performed an intensive sampling of multiple sites along the respiratory tract of healthy human individuals and observed low levels of bacterial diversity in the lower respiratory tract, but high levels of bacterial diversity in the URT^63^. Additionally, Huang *et al.*^64^ detected high diversity levels in the airway bacterial community of patients with chronic obstructive pulmonary disease^64^. Interesting a negative correlation was observed between the Shannon diversity index and bacterial load, indicating that when microbial colonization increases there is a corresponding reduction in microbial diversity. This finding is supported by Boutin *et al.*^65^, who reported a negative correlation between bacterial load and alpha-diversity index in the nasal cavity of human”^65^.

## Conclusions

In summary, this study demonstrated that the URT of Holstein dairy calves from 3 days to 35 days of life encompasses a highly rich and diverse bacterial community. Thirty genera were shared between all ages and health statuses and *Mannheimia, Mycoplasma, Moraxella* were the three most common bacterial genera detected in the calves URT. Our data supports the concept that *Mannheimia* and *Mycoplasma* are two dominant bacteria associated with pneumonia and otitis. Additionally, the genus *Moraxella* could play an important role in the pathogenesis of pneumonia and otitis, and the high neonatal bacterial load is a significant contributor to the development of pneumonia. These results provide an unprecedented understanding of the evolution of the bovine URT microbiome in pre-weaning calves and its association with RT diseases.

## Materials and Methods

### Ethics statement

This study was conducted on a commercial dairy farm located near Ithaca, New York, from November 2013 until February of 2014. The collection of nasal swabs samples from calves at days 3, 14, 28 and 35 of life was authorized by the farm owner. “The Animal Care and Use Procedures are produced and enforced to ensure the welfare of animals used in research and teaching at Cornell University. They ensure that high quality care is provided for all research animals, and that they are used in compliance with federal, state, and local regulations and guidelines”. Animal Care and Use Procedures were cared for according to the guidelines set by Dairy Cattle Husbandry (n^o^ 518)^66^. All experimental protocols using cattle were reviewed and approved by the Institutional Animal Care and use Committee of Cornell University (Protocol number: 2013-0076).

### Animals and facilities

Pregnant cows at stage 1 or 2 of parturition were transferred from the close-up free-stall barn into two maternity pens (400 m^2^ deep-bedded pens). After parturition, calves were removed from the maternity pen and placed into a newborn pen bedded with dry sawdust and heated with heating lamps during the winter months. Colostrum from multiparous and primiparous cows was pooled and used in the study. All calves were fed approximately 4 L of raw colostrum at once by an esophageal feeder (Oral Calf Feeder Bag with Probe, Jorvet) within 4 hours of birth.

Twice daily, newborn calves were allocated from the newborn pen to the calf barn. The calf barn was a greenhouse type of barn with positive ventilation and divided into 18 identical group-pens. Group-pens had a total area of 70 m^2^ and were bedded with straw bedding on top of a thin layer of dry composted manure. Steel gates divided the group pens, and calves were allocated by birth order into each pen until the pen was completely full (a total of 25 calves per pen). All calves remained in the same pen from day 1 of life until fully weaned (approximately 65 days). Birth weight and weight at weaning of all heifer calves were measured by farm employees using a Waypig 15, 62-inch digital scale (Waypig-15, Vittetoe Inc., Keota, IA).

Calves were fed ad-libitum acidified non-saleable milk. The feeding system was fully automated. Briefly, the acidification was performed inside a sealed stainless-steel tank where the non-saleable cold (5 °C) milk was mixed continuously with organic acid until pH 4.5 was reached. The acidified milk was kept for 72 hours inside the stainless-steel tank after the acidification process was finished. Then, the milk was directed to a smaller stainless-steel tank, which maintained the milk at a warm temperature and distributed it to the pens. To support the ad-libitum system, 6 nipples per pen were connected to the smaller tank and the acidified non-saleable milk was available from day 1 to day 55 of life, when a reduction of milk availability was initiated. All calves in this study were weaned by reducing the milk availability starting on day 55 until complete absence of acidified non-saleable milk at 65 days of life.

### Deep pharyngeal swab collection

A cohort of 174 Holstein heifer calves was selected randomly for this study. Deep pharyngeal swabs were performed on days 3, 14, 28, and 35 of life using a 20-cm DNA-free sterile swab (Puritan Medical Products, Guilford, ME) covered by a thin sterile plastic sheath. Prior to sampling, the selected calves were appropriately restrained and the nostril was cleaned using a paper towel. The plastic-covered sterile swab was inserted into the right nasal cavity at a depth of approximately 15 cm, the plastic sheet was then broken, exposing the swab to the URT mucosa, and a 360^o^ rotation was performed to better standardize sample collection. The swab was then retracted back into the plastic sheath and removed. The tip of the swab was placed inside a sterile plastic tube and labeled. Samples were kept on ice until they were transferred to the laboratory at Cornell Veterinary School and stored at −20 °C until further processing.

### Case definition

Pneumonia was defined when two or more of the following clinical signs were detected in a calf: cough, rectal temperature >39.5 °C, respiratory rate >40 breaths/min, increased cranioventral lung sounds or wheezes. Otitis was defined by observation of ear pain evidenced by head shaking, scratching or rubbing the ears, epiphora, ear droop, signs of facial nerve paralysis, with or without fever (rectal temperature >39.5 °C).

Two dedicated farm employees with over 10 years of experience and trained by Cornell University veterinarians (Ambulatory and Production Medicine Department), were responsible for overseeing the calf facility and making the initial detection of pneumonia and otitis. When farm employees detected animals that were displaying signs of disease such as depression, inappetence, dehydration, increased respiratory rate, or a head tilt (otitis) a full physical examination was carried out to determine the diagnosis of the disease. Once the farm employees examined the affected calves an experienced veterinarian member of the research team performed a second confirmatory physical examination. Calves diagnosed with pneumonia and/or otitis were treated according to standard farm protocol ((Resflor Gold, Merck Animal Health).”

### DNA extraction

Isolation of DNA from swabs of the URT was performed by adding 1.5 ml of DNA-free water into a 2-ml microcentrifuge tube containing a nasal swab sample, which was disrupted using a Mini-Beadbeater-8 (Biospec Products, Battersville, OK). Swabs were removed from the microcentrifuge tubes and the remaining liquid was centrifuged for 10 minutes at 13,000 rpm. The supernatant was discarded and the DNA was extracted from the pellet using the PowerSoil DNA Isolation Kit (MO BIO Laboratory Inc., Carlsbad, CA). DNA concentration and purity were evaluated using a NanoDrop ND-1000 spectrophotometer (NanoDrop Technologies, Rockland, DE) at wavelengths of 230, 260, and 280 nm.

### Quantitative PCR

In order to determine the total bacterial load of the URT samples, we cloned a plasmid containing the amplified V6 hypervariable region into TOP10 cells by using a Zero Blunt^®^ TOPO^®^ PCR cloning kit (Life Technologies, Darmstadt, Germany). Plasmid was purified with a QIAprep Spin Miniprep Kit (Qiagen, Valencia, CA) and quantified using Quant-iT™ PicoGreen^®^ and a dsDNA Broad Range Assay Kit (Life Technologies Corporation, Carlsbad, CA). Insertion was confirmed by agarose gel electrophoresis, and by sequencing the plasmid at the Cornell University Life Science Core Laboratories Center.

16S rRNA gene copy numbers were measured by quantitative PCR (qPCR) using Unibac primer (forward: 50-TGG AGC ATG TGG TTT AAT TCG A-30; reverse: 50-TGC GGG ACT TAA CCC AAC A-3)^65^,^67^. PCRs were performed in 15 μL volumes composed of 1X iQ^TM^Sybr Green Supermix (BIO-RAD Laboratories, Hercules, CA), 300 nM of each primer and 50 ng–5 pg of genomic DNA (or plasmid DNA standards). The thermal cycler conditions were as follows: denaturation at 95 °C for 3 min, 40 amplification cycles (95 °C for 10 s, 55 °C for 30 s) and two final steps at 95 °C for 1 min and 55 °C for 1 min followed by melting curve determination. All reactions were performed in duplicate using a MyiQ^TM^ Real-Time PCR Detection System (BIO-RAD Laboratories, Hercules, CA). Quantification of 16S rRNA target DNA was achieved by 10-fold serial dilutions ranging from 10^0^ to 10^7^ plasmid copies of the previously quantified plasmid standards. Plasmid standards and URT samples were run in duplicates. The average of the cycle threshold value was used for calculation of the total bacterial load.

### PCR amplification of the V4 hypervariable region of bacterial 16S rRNA genes

The 16S rRNA gene was amplified by PCR from individual metagenomic DNA samples from the URT using barcoded primers. For amplification of the V4 hypervariable region of the bacterial/archaeal 16S rRNA gene, primers 515F and 806R were used according to previously described methods and optimized for the Illumina MiSeq platform^68^. The Earth Microbiome Project (http://www.earthmicrobiome.org/)^69^ was used to select 140 different 12-bp error-correcting Golay barcodes for the 16S rRNA gene PCR, as previously described^68^. The 5′-barcoded amplicons were generated in triplicate using 12–300 ng of template DNA, 1X EconoTaq^®^ Plus Green Master Mix (Lucigen^®^, Middleton, WI) and 10 μM of each primer. The PCR conditions for amplification of the 16S rRNA gene included an initial denaturing step of 94 °C for 3 min followed by 35 cycles of 94 °C for 45 s, 50 °C for 1 min and 72 °C for 90 s and a final elongation step of 72 °C for 10 min. Before sequencing, replicate amplicons were pooled and purified with a QIAquick PCR Purification Kit (Qiagen, Valencia, CA) and visualized by electrophoresis using 1.2% (wt/vol) agarose gels stained with 0.5 mg/ml ethidium bromide. Blank controls in which no DNA was added to the reaction were performed. Purified amplicon DNA was quantified using Quant-iT™ PicoGreen^®^ and a dsDNA Broad Range Assay Kit (Life Technologies Corporation, Carlsbad, CA).

### Sequencing, bioinformatics, and statistical analysis

Aliquots of URT amplicon samples were standardized to the same concentration and pooled into 5 different sequencing runs according to individual barcode primers for the 16S rRNA gene. Final equimolar libraries were sequenced using the MiSeq reagent kit v2 (300 cycles) on the MiSeq platform (Illumina, Inc., San Diego, CA). The generated 16S rRNA gene sequences were processed through the open source software pipeline Quantitative Insights Into Microbial Ecology (QIIME) version 1.7.0-dev^68^. Sequences were filtered for quality using established guidelines^70^. Sequences were binned into OTUs based on 97% identity using UCLUST^71^ against the Greengenes reference database^72^, May 2013 release. Low-abundance clusters were filtered and chimeric sequences were removed using USEARCH^71^. The representative sequences for each OTU were compared against the Greengenes database for taxonomy assignment, and only full-length, high-quality reads (−r = 0) were used for analysis. Additionally, we generated a species-level OTU table using the MiSeq Reporter Metagenomics Workflow. The MiSeq Reporter classification is based on the Greengenes database (http://greengenes.lbl.gov/) and the output of this workflow is a classification of reads at multiple taxonomic levels: kingdom, phylum, class, order, family, genus, and species.

Shannon and Chao1 indexes output were generated by QIIME pipeline. Before estimating the Shannon and Chao1 indexes, all sample libraries were rarefied to an equal depth of 10,000 sequences using QIIME. Chao1 and Shannon indexes, total number of reads, and the log of the 16S rDNA copy number (total bacterial load) were analyzed using repeated measures ANOVA by general linear models fitted in JMP Pro 11 (SAS Institute Inc., Cary, NC). Dunnett’s multiple comparisons procedure was performed to compare the mean number of reads, Shannon index and Chao 1 index of each disease status (otitis, pneumonia, and pneumonia-otitis combined) against the healthy samples within each day of data collection (days 3, 14, 28, and 35).

Correlations between total bacterial load and alpha-diversity indexes (Shannon and Chao 1 indexes) were assessed using simple linear regression in JMP Pro 11 software (SAS Institute Inc.).

The relative abundances of microbial phyla and genera types in URT samples of calves at ages 3, 14, 28, and 35 within each health status were compared using general linear models fitted in JPM Pro 11 (SAS Institute Inc.). Dunnett’s multiple comparisons procedure was used to compare the mean relative abundance of the most abundant bacterial phyla and the genera of each disease status (otitis, pneumonia, and pneumonia-otitis combined) against the healthy samples within each day of data collection (days 3, 14, 28, and 35). Differences with a value of *P* ≤ 0.05 were considered significant and those with a value of 0.05 < P ≤ 0.10 were considered tendencies.

Descriptive statistics for birth weight (BW) and average daily gain (ADG) were determined according to health status by using a general linear model (ANOVA) with JMP Pro 11 (SAS Institute Inc.). In total, 174 calves were enrolled in this study. Number of calves, disease incidence, mortality, BW and ADG (calculated by subtracting BW from the weaning weight and then dividing by days of life at weaning) during the pre-weaning period are presented in Table 1. Average age (days in life) at first diagnosis of pneumonia, otitis and pneumonia_otitis combined was assessed by using Distribution platform offered by JMP Pro 11 (SAS Institute Inc.).

## Additional Information

**How to cite this article**: Lima, S. F. *et al.* The upper respiratory tract microbiome and its potential role in bovine respiratory disease and otitis media. *Sci. Rep.* **6**, 29050; doi: 10.1038/srep29050 (2016).

## Supplementary Material

Supplementary Information

## Figures and Tables

**Table 1:** Descriptive overview of the calves selected and enrolled in the study.

*n*	Healthy	Pneumonia	Otitis	Pneumonia and Otitis
	64	37	62	11
Incidence (%)	36.8	21.3	35.6	6.3
Mortality (%)	1.15	0.57	1.72	0.57
Birth weight (kg)	38.05 (0.51)	38.36 (0.67)	38.52 (0.53)	36.16 (1.23)
ADG^1^ (g/day)	659.4 (23.3)	633.9 (30.2)	577.4 (24.4)	564.6 (60.7)
ADL^2^	–	22.5 (1.3)	24 (0.9)	19.7 (2.0)

^1^ADG = Average daily gain was calculated by subtracting birth weight from the weaning weight and then dividing by days of life at weaning.

^2^ADL = Average days of life at first diagnosis.

**Table 2:** Correlation between Shannon diversity index and bacterial load in each heath status investigated.

Health status	Correlation	*P* - values
Healthy	−0.53	<0.0001
Pneumonia	−0.24	0.02
Otitis	−0.35	<0.001
Pneumonia-otitis combined	−0.48	0.01

**Table 3:** Descriptive statistics of the 30 most abundant bacterial genera.

Genera	3 days		14 days		28 days		35 days	
	P^1^	MRA^2^	P	MRA	P	MRA	P	MRA
*Mannheimia*	100%	3.1	100%	24.9	100%	15.6	100%	15.4
*Mycoplasma*	100%	2.9	100%	10.8	100%	18.2	100%	15.5
*Moraxella*	100%	9.1	100%	18.4	99%	2.6	100%	4.8
*Psychrobacter*	100%	13.5	100%	8.4	99%	3.1	100%	7.0
*Pseudomonas*	100%	4.0	100%	13.1	100%	5.5	99%	1.2
*Acinetobacter*	100%	5.3	100%	1.7	100%	5.2	100%	3.6
*Cellulomonas*	98%	0.2	84%	0.1	98%	5.2	100%	5.2
*Bacteroides*	100%	3.3	99%	1.2	99%	2.3	100%	4.0
*Escherichia*	100%	4.2	99%	0.6	100%	2.9	99%	0.3
*Corynebacterium*	100%	2.5	99%	0.3	99%	2.1	100%	2.2
*Fusobacterium*	100%	1.1	100%	0.7	99%	1.9	100%	1.5
*Pasteurella*	100%	0.7	99%	2.2	99%	0.8	99%	0.6
*Pedobacter*	100%	1.1	99%	0.2	98%	2.3	100%	1.2
*Streptococcus*	100%	1.8	100%	1.5	99%	0.7	99%	0.5
*Serratia*	100%	2.6	99%	0.4	100%	1.4	99%	0.3
*Prevotella*	100%	1.0	99%	0.4	99%	0.8	100%	1.8
*Staphylococcus*	100%	1.1	99%	0.2	100%	1.8	100%	0.7
*Ruminococcus*	100%	1.3	99%	0.3	99%	0.7	100%	1.2
*Candidatus Blochmannia*	100%	0.9	100%	0.3	100%	1.1	100%	1.1
*Porphyromonas*	100%	1.3	99%	0.2	99%	0.4	100%	1.4
*Clostridium*	100%	1.9	100%	0.2	99%	0.4	100%	0.9
*Blautia*	100%	1.0	100%	0.2	99%	0.9	100%	1.2
*Brenneria*	100%	0.9	99%	0.8	96%	0.1	98%	0.5
*Gallibacterium*	100%	1.0	96%	0.5	99%	0.7	100%	0.4
*Stenotrophomonas*	99%	0.1	95%	0.1	99%	1.5	100%	0.4
*Treponema*	100%	0.9	100%	0.5	98%	0.2	99%	0.7
*Sphingobacterium*	100%	0.5	100%	0.5	99%	0.8	99%	0.4
*Aggregatibacter*	85%	0.1	99%	1.0	98%	0.1	97%	0.2
*Aerococcus*	99%	0.7	97%	0.2	99%	0.5	100%	0.4
*Flavobacterium*	100%	0.5	96%	0.1	99%	0.6	100%	0.5

^1^P = Percentage of study calves in which the indicated genus was detected at the given age at sample collection.

^2^MRA = Mean relative abundance of the respective bacterial genera at the given postnatal age.

**Table 4:** The 20 most abundant genera detected in the URT and the correspondent abundance according to each heath conditions (healthy, otitis, pneumonia, pneumonia and otitis combined) and postnatal age (3, 14, 28 and 35 days of life).

Genera	3 days				14 days				28 days				35 days			
	^1^H	^2^O	^3^P	^4^P&O	^1^H	^2^O	^3^P	^4^P&O	^1^H	^2^O	^3^P	^4^P&O	^1^H	^2^O	^3^P	^4^P&O
*Mannheimia*	2.4	3.4	4.5	0.7	19.8^a^	25.5^ab^	31.7^b^	27.6^ab^	10.0^a^	13.7^a^	23.7^b^	32.1^b^	15.2	18.3	12.5	9.9
*Mycoplasma*	3.0	3.0	2.9	3.0	12.1	9.5	9.9	13.3	11.0^a^	20.8^b^	21.8^b^	33.3^c^	15.8	15.8	13.7	17.7
*Moraxella*	8.0	11.0	8.9	5.1	13.5^a^	20.2^b^	21.9^b^	24.8^b^	1.9	2.1	4.3	4.3	3.2^a^	4.4	7.2^b^	8.4
*Psychrobacter*	12.8	14.4	13.5	11.9	7.8	8.3	10.9	3.5	2.8	2.4	4.5	4.3	6.4	7.8	6.7	6.2
*Pseudomonas*	4.0	3.7	4.3	4.4	19.9^a^	12.4^b^	5.3^b^	4.0^b^	8.5^a^	6.1^a^	0.9^b^	0.4^b^	1.3	1.1	1.1	0.9
*Acinetobacter*	5.8	3.9	6.3	6.7	2.6	1.4	0.9	1.2	6.0	4.7	6.0	1.0	4.9	3.1	2.2	3.0
*Cellulomonas*	0.1	0.1	0.3	0.2	0.1	0.1	0.0	0.1	2.8	6.1	8.0	5.5	5.9	5.0	3.8	7.5
*Bacteroides*	3.1	3.5	2.6	5.6	1.0	1.4	1.2	1.1	2.3	2.5	2.2	1.5	3.7	3.9	4.8	3.9
*Escherichia*	4.7	4.0	3.9	3.3	0.5	0.9	0.3	0.5	4.5^a^	3.3^a^	0.2^b^	0.1^b^	0.3	0.3	0.3	0.1
*Corynebacterium*	2.4	2.4	3.1	1.9	0.4	0.3	0.2	0.4	2.9	2.1	1.0	0.4	2.6	1.9	1.9	2.2
*Fusobacterium*	1.2	1.3	0.8	1.5	0.6	0.7	0.8	0.5	0.9	2.4	2.8	0.9	1.6	1.7	1.1	0.5
*Pasteurella*	1.5	0.2	0.3	0.2	3.3^a^	1.2^b^	2.6^ab^	0.7^ab^	1.4	0.4	0.7	0.4	0.4	0.9	0.4	0.2
*Pedobacter*	1.1	1.0	1.2	1.7	0.2	0.2	0.1	0.1	2.6	2.8	1.2	1.6	1.1	1.1	1.6	1.3
*Streptococcus*	1.9	1.9	1.6	1.5	1.4	1.1	2.2	2.1	0.5	0.9	0.7	0.4	0.5	0.5	0.5	0.6
*Serratia*	3.0	2.5	2.4	2.2	0.4	0.5	0.2	0.3	2.2	1.7	0.1	0.1	0.3	0.2	0.2	0.1
*Prevotella*	0.9	1.1	0.7	1.6	0.4	0.5	0.2	0.6	1.1	0.7	0.5	0.2	1.8	1.4	2.6	1.7
*Staphylococcus*	1.3	0.9	1.2	1.1	0.2	0.2	0.1	0.1	2.3	2.1	0.7	0.2	0.7	0.7	0.8	0.6
*Ruminococcus*	1.2	1.4	1.4	1.8	0.3	0.4	0.1	0.3	0.8	0.6	0.7	0.4	0.9	1.1	1.7	1.6
*Candidatus Blochmannia*	0.9	0.9	0.9	1.0	0.2	0.3	0.2	0.4	1.5	1.1	0.7	0.6	1.1	1.0	1.3	1.1
*Porphyromonas*	1.2	1.6	1.1	1.8	0.1	0.3	0.1	0.3	0.5	0.4	0.3	0.2	1.2	1.5	1.5	1.3

^1^H = Healthy; ^2^O = Otitis; ^3^P = Pneumonia; ^4^P&O = Pneumonia and otitis combined.
