# Supplementary material for: The upper respiratory tract microbiome and its potential role in bovine respiratory disease and otitis media
Source: Sci Rep. 2016 Jul 1;6:29050. doi: 10.1038/srep29050 (PMC4929571; doi:10.1038/srep29050)

The upper respiratory tract microbiome and its potential role in bovine respiratory disease and otitis media

Svetlana F. Lima[1], Andre Gustavo V. Teixeira[1], Catherine H. Higgins[1], Fabio S. Lima[2], Rodrigo C. Bicalho[1]*

[1]Department of Population Medicine and Diagnostic Sciences, College of Veterinary Medicine, Cornell University, Ithaca, New York, USA

[2]Department of Veterinary Clinical Medicine, College of Veterinary Medicine, University of Illinois, Urbana, Illinois, USA

*Address correspondence to Rodrigo Carvalho Bicalho, rcb28@cornell.edu

Supplemental 1: Correlation between Shannon diversity index and bacterial load of the neonatal calf upper respiratory tract. The bacterial load was measured via proxy of the number of 16S rRNA genes. Samples from all sampling time points are represented.

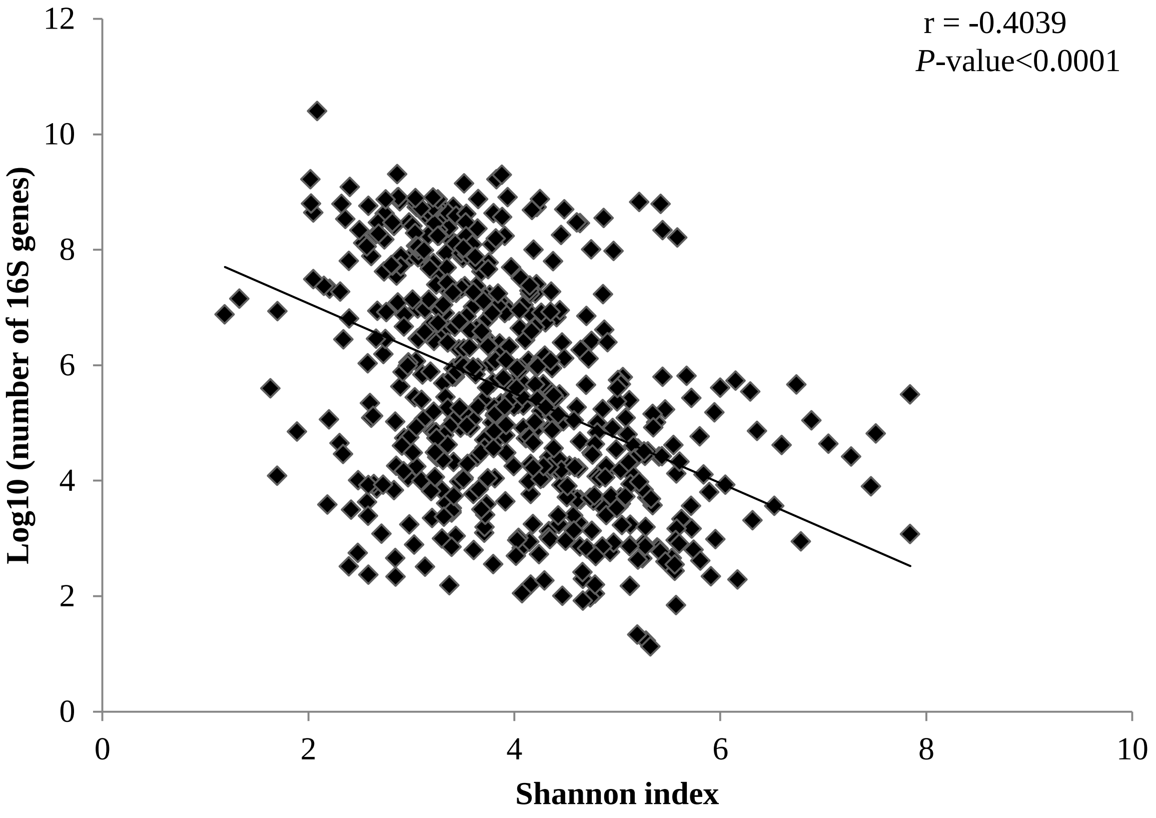

Supplemental 2: Mean relative abundance of the genus *Pseudomonas* according to postnatal age at sample collection (3, 14, 28, 35 days) and health status (healthy = green, otitis = orange, pneumonia = pink, and pneumonia-otitis combined = red). Error bars are positioned around the means and represent the standard error of the mean, and contours show regions of data density. Dunnett's multiple comparison procedure was used to compare each disease status (otitis, pneumonia, and pneumonia-otitis combined) against the status "healthy" within each sample collection age. [a,b] different superscripts on a series of data points indicate a significant difference ($P < 0.05$) between the respective health status categories within each age group sampled.

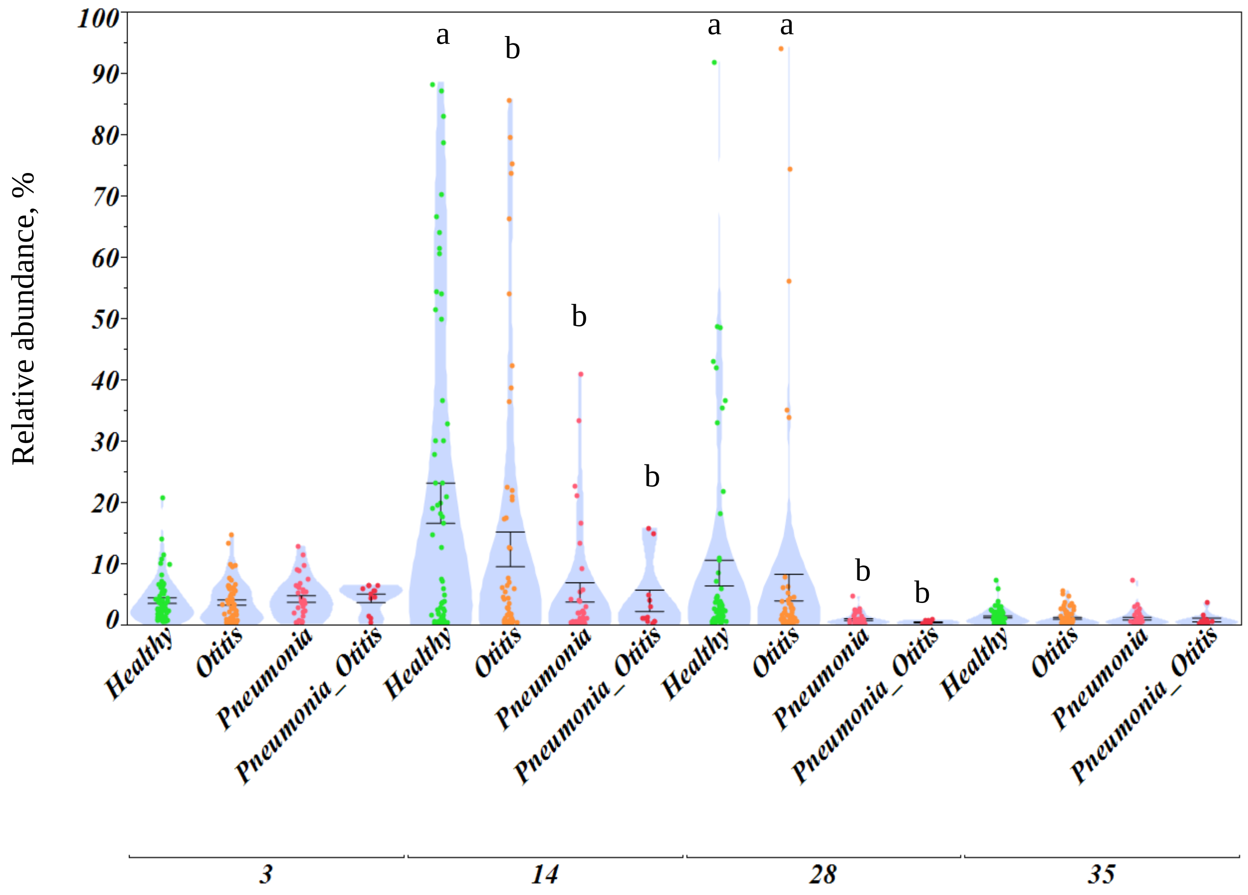

Supplemental 3: Mean relative abundance of the genus *Escherichia* according to postnatal age at sample collection (3, 14, 28, 35 days) and health status (healthy = green, otitis = orange, pneumonia = pink, and pneumonia-otitis combined = red). Error bars are positioned around the means and represent the standard error of the mean, and contours show regions of data density. Dunnett's multiple comparison procedure was used to compare each disease status (otitis, pneumonia and pneumonia-otitis combined) against the status "healthy" within each sample collection age. [a,b] different superscripts on a series of data points indicate a significant difference ($P < 0.05$) between the respective health status categories within each age group sampled.

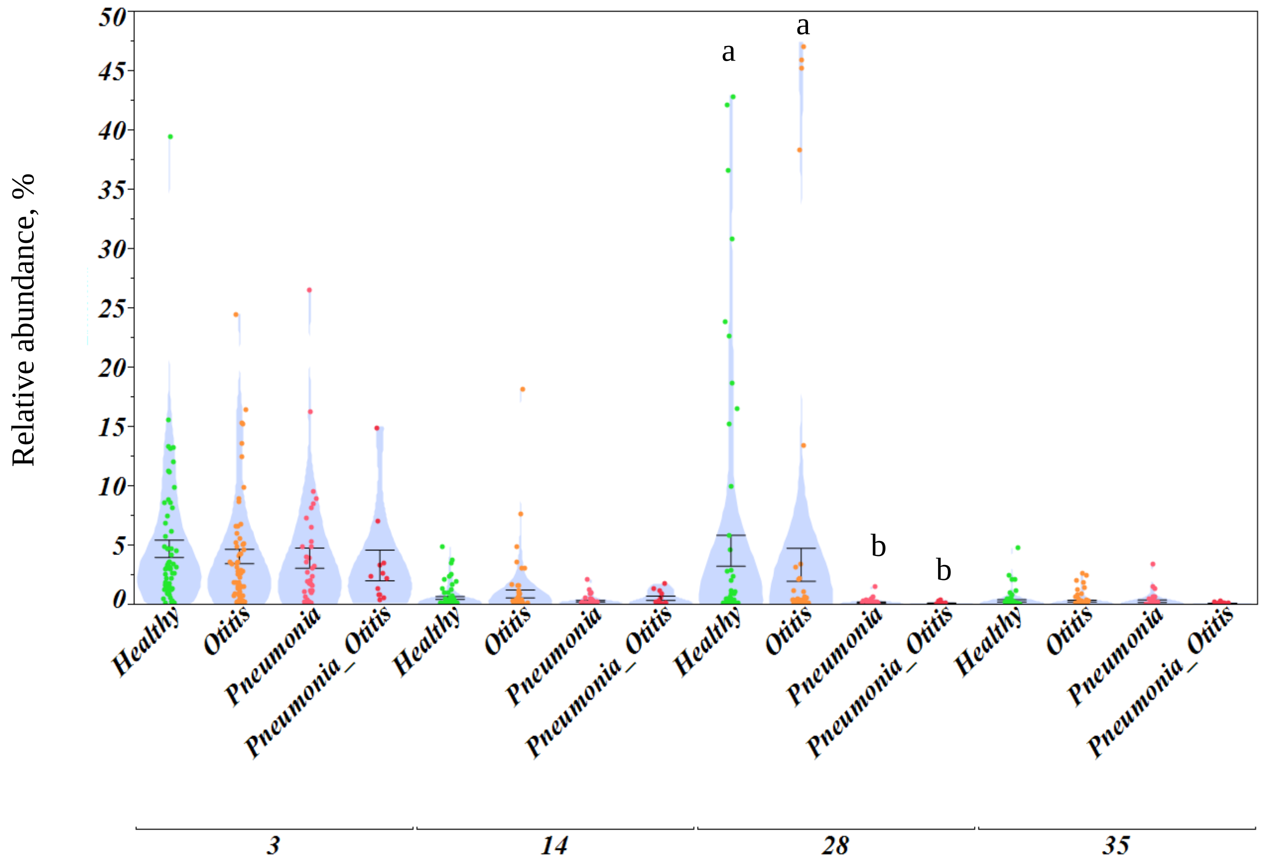

Supplemental 4: Relative abundance of the genus *Corynebacterium* by health status (healthy = green, otitis = orange, pneumonia = light red, and pneumonia-otitis = dark red) and postnatal age at sample collection (3, 14, 28, and 35 days). Error bars are positioned around the means and represent the standard error of the mean, and contours show regions of data density. Dunnett's multiple comparison procedure was used to compare each disease status (otitis, pneumonia and pneumonia-otitis combined) against the status "healthy" within each sample collection age. [a,b] different superscripts on a series of data points indicate a significant difference (P < 0.05) between the respective health status categories within each age group sampled.

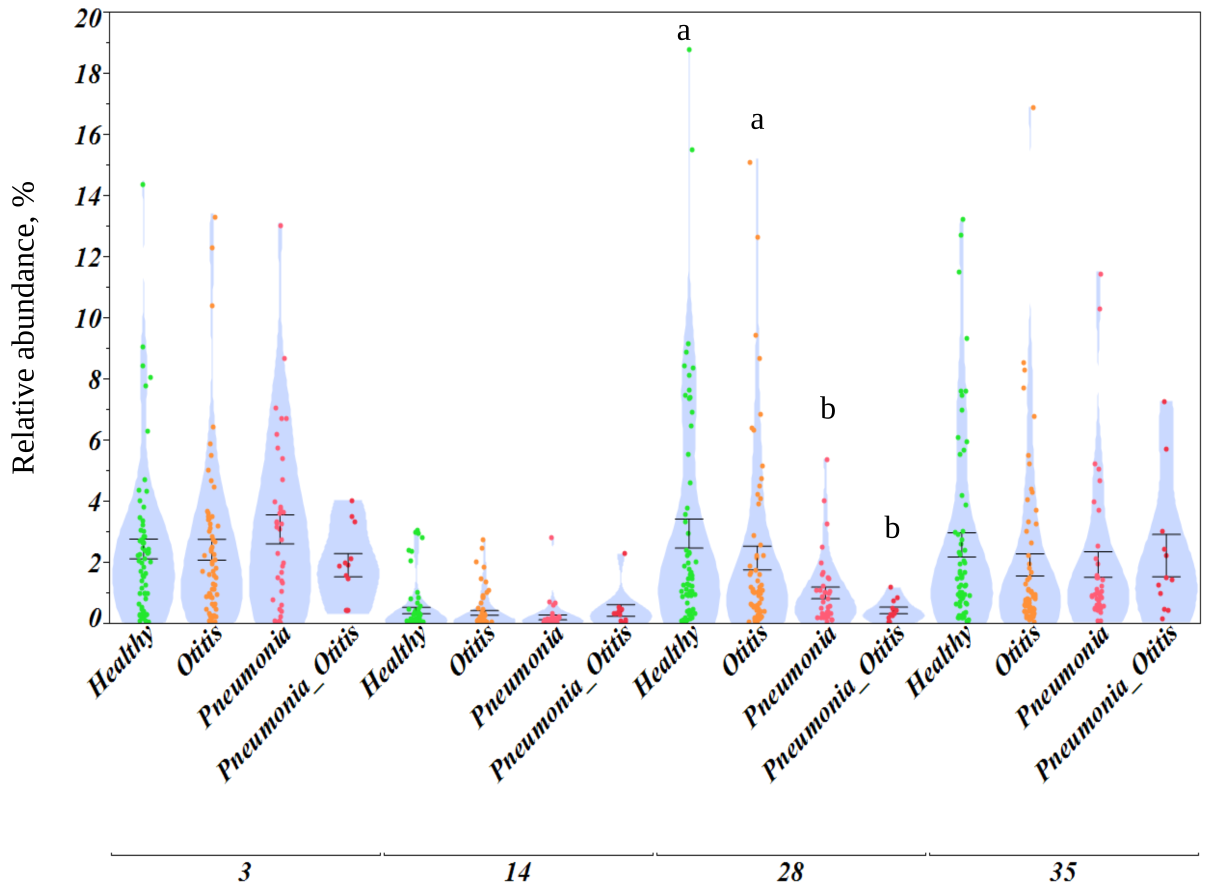

Supplement: Supplementary Information [file srep29050-s1.pdf]
